# Margin evaluation of translational and rotational set-up errors in intensity modulated radiotherapy for cervical cancer

**DOI:** 10.1186/s40064-016-1796-2

**Published:** 2016-02-24

**Authors:** Xiang Zhang, Guo-ping Shan, Ji-ping Liu, Bin-bing Wang

**Affiliations:** Department of Gynecologic Radiation Oncology, Zhejiang Cancer Hospital, Hangzhou, 310022 China; Department of Radiation Physics, Zhejiang Cancer Hospital, Hangzhou, 310022 China; Zhejiang Key Laboratory of Radiation Oncology, Hangzhou, 310022 China

**Keywords:** Rotational set-up errors, Image-guided radiotherapy, Cervical cancer, Intensity modulated radiotherapy

## Abstract

A clinical target volume (CTV) to planning target volume (PTV) margin recipes was routinely used to ensure dose was actually delivered to target for all (most) patients. Currently used margin recipes were associated with only translational set-up errors in radiotherapy. However, when set-up errors extended to six-degree (6D) scope (three translational and three rotational set-up errors), margin recipe should be re-evaluated. The purpose of this study was to investigate dosimetric changes of targets (both CTV and PTV) coverage when 6D set-up errors were introduced and testify the practicability of currently used margin recipe in radiotherapy. A total number of 105 cone beam computer tomography scans for ten patients with cervical cancer were derived prior to treatment delivery and 6D set-up errors were acquired with image registration tools. Target coverage was evaluated retrospectively for 6D set-up errors introduced plan with 6 mm CTV to PTV margin. Target coverage of PTV showed significant decreases (3.3 %) in set-up errors introduced plans compared with original plans. But CTV coverage was not susceptible to these set-up errors. A tendency of coverage decrease for PTV along with distance away from treatment was testified, from −0.2 to −6.2 %. However, CTV seems changed less, from −0.2 to −0.8 %. The result indicate that a CTV to PTV margin of 6 mm was sufficient to take into account 6D set-up errors for most patients with cervical cancer. Future research suggests a smaller margin to further improve both tumor coverage and organs at risk sparing.

## Background

External beam radiotherapy (EBRT) followed by brachytherapy is the standard treatment for cervical cancer, especially for advanced disease. Intensity modulated radiotherapy (IMRT) is increasingly used for EBRT of cervical cancer for its satisfying dose distribution on a plan to ensure target dose and limit normal tissue irradiation (Chen et al. 2007; Brixey et al. 2002; van de Bunt et al. 2006; Ahamad et al. 2005). However, on the other hand, the more accurate dose distribution of IMRT, the more precise treatment procedure is needed. Positional uncertainties and organ motion are two major factors contribute to treatment accuracy in EBRT (van Herk 2004; Beard et al. 1996; Antolak et al. 1998). Safety margin from CTV to PTV is a standard approach to ensure that the delivery region is adequate despite uncertainties in treatment.

Image guided radiotherapy (IGRT) provides an excellent solution to quantify and correct for patient set-up errors in modern radiotherapy (Zeidan et al. 2007; Mackie et al. 2003; Letourneau et al. 2005; Verellen et al. 2003). The IGRT equipment mounts an x-ray tube and a flat panel detector, CBCT, on linear accelerator. Patients have daily CBCT scan prior to treatment delivery. By sort of image registration method, one can estimate patient pose difference in the treatment room from the planning CT. With this advanced imaging techniques, 6D set-up errors (3 translational and 3 rotational, pitch, roll and yaw) can be detected and derived. Unfortunately, rotational set-up errors are not routinely corrected because most couches currently available can only correct one degree of these rotational movements.

The impact of rotational set-up errors is an attractive issue for researchers (van Herk 2004; Guckenberger et al. 2006; Kim et al. 2008; Lim et al. 2009; Peng et al. 2013). Cervical cancer target has the characteristic that it always has large cranio-caudal extension. Therefore, the target coverage is more susceptible to rotational movements especially in pitch rotation. There are some studies reported that larger discrepancies generated by pitch errors were found in cervical cancers (Laursen et al. 2012; Quint et al. 2001; Kaiser et al. 2006). A CTV to PTV margin recipes (2.5 SD of systematic translational set-up errors plus 0.7 SD of random translational set-up errors) introduced by van Herk et al. is Commonly used regardless of rotational set-up errors and shape of target (Lips et al. 2009). Whether these discrepancies are significant in dosimeteric for cervical cancers, should be evaluated carefully.

Rotational movements can be compensated by the method of using gantry and collimator rotations to correct for target movements (Yue et al. 2006). It is an alternative method of applying 6D repositioning device. This is also an ideal method to reestablished accumulated dose of errors introduced plan in treatment planning system (TPS). The purpose of this study is to evaluate the impact of correct rotational set-up errors in dosimeteric with clinical obtained data from multi-fractions.

## Methods

### Patient selection and plan

Ten cervical cancer patents undergoing definitive radiotherapy in Zhejiang Cancer Hospital during January 2012 and June 2012 were retrospectively reviewed. Planning CT scans were performed with Philips CT-sim scanner with 5-mm slice thickness (Philips Medical System, Eindhoven, Netherlands). Patients were treated in supine position and placed on room laser system according to markers on body. All patients used customized immobilized device, a thermoplastic mask, to eliminate set-up errors between each fractions.

All treatment planning was customized using IMRT to obtain high conformity dose distribution. This study used a CTV-PTV uniform margin recipe of 6 mm according to van Herk margin recipe and our clinical protocol. For patients included in this study, prescription dose range was 45 Gy in 25 treatment fractions. For each patient, dose was prescribed to cover 95 % of PTV. A beam arrangement consisting of 7 coplanar beams was used.

IMRT was delivered with 10 MV by a linear accelerator (Elekta Oncology Systems, Crawley, UK). None of these patients received para-aortic treatment.

### Set-up errors detection

CBCT was derived prior to treatment fraction and a total number of 105 CBCT image sets were acquired for 10 patients. The selected scan parameter was 125 kVp and 1.6mAs per projection. Automatic rigid volumetric image registration of the CBCT to the planning CT was performed on XVI (Elekta Oncology Systems, Crawley, UK) for the purpose of correcting any misalignment of body position in treatment. Bony match registration method was performed on all bony information available. The registration result reported residual set-up errors in six degrees, three translations T(x, y, z) and three rotations R(θx, θy, θz). Here X was in lateral direction, Y was in anterior-posterior direction, Z was in superior-inferior direction, in treatment machine coordinate.

### Equivalent beam parameters

Translational movements can be corrected by shifting treatment couch laterally, longitudinally, and vertically in most treatment machines but rotation movements can only be eliminated by robot treatment couch. However, the robot six-degree couch is not widely equipped. In this study, equivalent beam parameters (gantry, collimator, couch angles) were calculated to compensate the patient treatment position rotation under rigid body assumption. For example, rotation in z-axis can be compensated by inverse rotation of gantry, rotation in y-axis can be compensated by collimator and couch rotation, rotation in x-axis can be eliminated by gantry, couch and collimator simultaneously. An in-house developed program used the algorithm (Yue et al. 2006), was coded to calculate the equivalent gantry, collimator, couch angles under rigid body assumption to compensate the patient treatment position rotation which can not be corrected. With this method, the rotational set-up errors can be equivalently corrected by rotating gantry/collimator/couch while keeping the patient position rather than adjust patient position by using robot treatment couch.

### Accumulation of delivered dose

For each patient, errors introduced plans were generated by shifting iso-center position and/or changing gantry, collimator and couch angles (see 2.3). The translational errors of (x, y, z) and rotational errors of (θx, θy, θz) were used to calculate the corresponding errors introduced plans. A total number of 105 errors introduced plans were created for translation only (Tonly) plans and translation plus rotation (TplusR) plans, respectively. The cumulative dose was calculated by multiply a weight equally for each investigated fractions

$$ D_{Cum} = \sum\limits_{n}^{i = 1} {\frac{n}{N}} D_{i} $$

where $D_{i}$ is dose distribution for $i$ th fraction. $n$ is the total number of scanning for one patient,$N$ is the total number of fraction, here $N = 25$.

### Dosimetric comparison

The three-dimensional (3D) dose distributions of reference plans (original plans) were compared with two types of errors introduced plans, Tonly plans and TplusR plans. The V_100_ is defined as the target volume that received 100 % of the prescription dose. All dosimetric parameters V_100_ for CTV and PTV were compared with relevant values obtained from original plan dose distribution and differences accumulated plan dose distribution for each patient. To estimate the value of rotation corrections, we further compared the differences in the dose distribution obtained with TplusR compared with those obtained with Tonly.

Cervical cancer target always has large cranio-caudal extension. Therefore, rotational uncertainties may potentially cause target displace in particular in the regions furthest away from the treatment center. In this study, targets of CTV and PTV were divided into several sections by distance away from treatment center in Z direction as follows: (1) volume of PTV within 4 cm in Z direction; (2) volume of PTV within 7 cm but larger than 4 cm in Z direction; (3) volume of PTV larger than 7 cm in Z direction. The target dose coverage dosimetric parameter V_100_ for each CTV and PTV section was calculated to evaluate the dose distribution affected by target displace particularly in the regions furthest away from the treatment center.

### Statistics and analysis

The paired-samples *t* test was used on differences between groups to determine whether correction strategy Tonly and TplusR is valuable, with a threshold of 0.05 for significance. Analyses were performed using SPSS 17.0 statistical software package (SPSS Inc., Chicago, IL, USA).

## Results

### Translational and rotational set-up errors

**Fig. 1:**
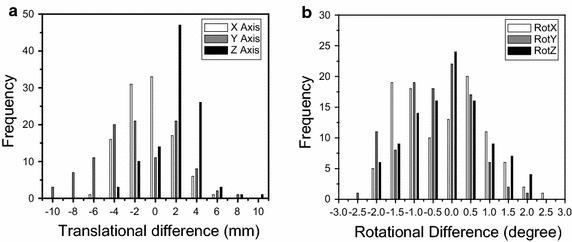
**a** Distribution of translational set-up errors in X, Y, Z axes and **b** rotational set-up errors around X (pitch), Y (roll), Z (yaw) axes for 105 CBCT scans

**Fig. 2:**
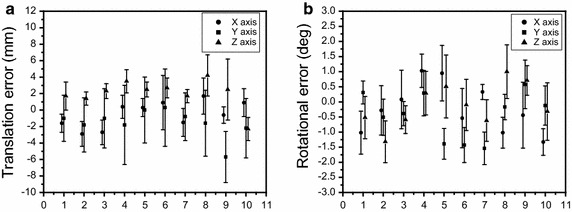
**a** The average and standard deviation of translational difference in 3 axes for each patient and **b** the average and standard deviation of rotations around 3 axes for each patient

Of 105 observations for the 10 patients with cervical cancer, distances from cranial to caudal for CTV and PTV were 19.9 ± 2.3 and 20.9 ± 2.3 cm, respectively. The obtained translational and rotational set-up errors, given in the format of mean ± standard deviation (SD), were X = −0. 6 ± 2.3 mm (range −5.1 to 5.3 mm), Y = −1.6 ± 3.9 mm (range −10 to 8.3 mm) and Z = 2.0 ± 2.4 mm (range −4.7 to 10 mm), RX = −0.2° ± 1.1° (range −2° to 2.3°), RY = −0.4° ± 0.9° (range −2.4° to 1.9°), RZ = −0.1° ± 1° (range −2° to 2.2°). Figure 1a, b show the distribution of translational set-up errors in X, Y, Z axes and rotational set-up errors around X (pitch), Y (roll), Z (yaw) axes as a group of 105 6D registrations for 10 patients with cervical cancer. Figure 2a shows the average and standard deviation of translational difference in 3 axes for each patient. Figure 2b shows the average and standard deviation of rotations around 3 axes for each patient involved in this study.

Translational shifts in Y direction larger than 6 mm occurred in a considerable number of treatment fractions in 15 of 105 observations. Whereas, translational shifts in X and Z resulted in such shifts were only 0 and 3 fractions, respectively.

Roll and yaw were rarely significant factors in the set-up accuracy as only a single fraction had rotations larger than 2°, respectively. Pitch was the most frequent source of residual rotational errors and resulted in rotation exceeding 2° in 3 fractions.

### Target coverage

**Fig. 3:**
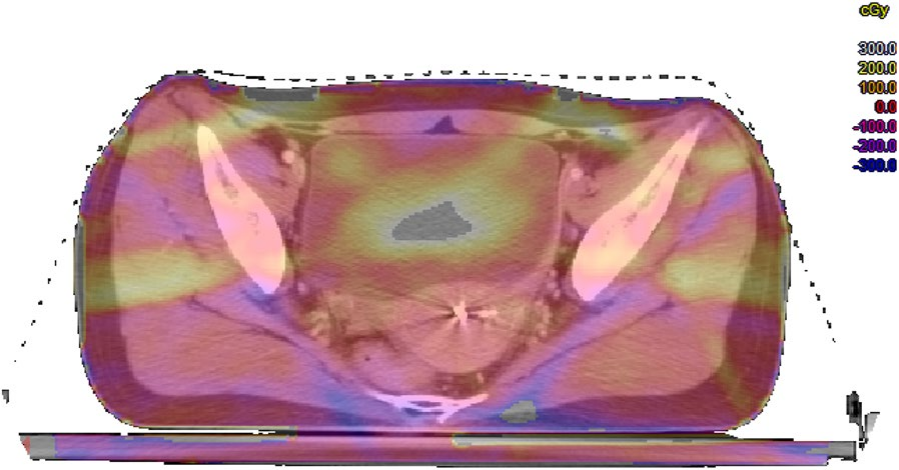
Example for total dose deviation of two plans

Total dose deviation of two plans, original plan and error introduced plan, was calculated for each patient. Difference between these two plans were show in Fig. 3. Percentage of V_100_ for all patients and investigated targets are shown in Table 1. For the original plan and errors introduced plan (TplusR) dose distribution, PTV V_100_ decreased 3.3 %. Deviations were statistically significant (p < 0.05) (Table 1). However, deviation in V100 % for CTV was not considerable (average 0.3 %, p = 0.03).

**Table 1:** The coverage difference changed of CTV and PTV in the clinic (in percentage)

	CTV V_100_			PTV V_100_		
	Original plan	TplusR	Tonly	Original plan	TplusR	Tonly
Minimum	99.08	98.67	98.47	95.01	89.85	90.7
Maximum	99.92	99.79	99.83	95.31	95.01	95.03
Mean	99.46	99.21	99.29	95.17	91.91	92.51
SD	0.08	0.14	0.15	0.01	2.56	2.30

**Table 2:** The coverage difference changed of each CTV section in the clinic (in percentage)

V_100_	CTV < 4 cm		4 cm < CTV < 7 cm		CTV > 7 cm	
	Original plan	T + R	Original plan	T + R	Original plan	T + R
Minimum	99.03	98.89	97.44	97.89	95.80	95.41
Maximum	100.00	99.94	99.95	99.96	99.92	99.83
Mean	99.68	99.49	99.12	99.07	99.22	98.46
SD	0.08	0.12	0.55	0.58	1.64	2.65

For each sections of PTV, V_100_ obtained with TplusR decreased with increasing distance away from treatment center, as expected. The observed mean differences between TplusR delivery and original plan in PTV > 7 cm, 4 cm < PTV < 7 cm and PTV < 4 cm, as derived from Nucletron Oncentra (Nucletron, Veenendaal, the Netherlands) treatment planning system, were −6.2 % (p = 0.0007), −2.5 % (p = 0.0002) and −0.2 % (p = 0.01), respectively (Table 3). For each section of CTV, differences between original plan and TplusR were hardly noticeable −0.8 % (p = 0.03), −0.1 % (p = 0.44), −0.2 % (p = 0.01) (Table 2).

**Table 3:** The coverage difference changed of each PTV section in the clinic (in percentage)

V_100_	PTV < 4 cm		4 cm < PTV < 7 cm		PTV > 7 cm	
	Original plan	T + R	Original plan	T + R	Original plan	T + R
Minimum	99.03	98.89	92.21	89.15	89.76	83.05
Maximum	100	99.94	97.5	95.21	96.06	92.15
Mean	99.68	99.49	94.91	92.39	93.97	87.81
SD	0.08	0.12	2.64	4.64	4.09	12.98

### Rotation correction

The increased dose coverageΔV_100_ obtained with TplusR and Tonly were compared in this study. Results show no significantly different when rotational set-up errors were correct before treatment. The results for CTV and PTV were0.08 % (p = 0.14) and 0.60 % (p = 0.02), respectively. Three of ten patients had PTV dose coverage boost larger than 1 % (range 1.1–2.4 %). Only one of ten patients had CTV dose coverage boost of 0.66 % (with PTV dose coverage boost 2.4 %) who suffered two fractions of rotational set-up errors larger than 2° (1 in yaw 2.2° and 1 in pitch 2.3°). The result implying that the effect of rotation correction seems to be very small to improve coverage on CTV and modestly on PTV.

## Discussions

Rotational errors were often considered to result in compromised target coverage. Previous margin recipe was determined by applying a common margin recipe according to translational set-up errors only in mostly institutions. It is necessary to evaluate the importance of correct rotational set-up errors when we got the fully scope of 6D set-up errors with CBCT and image registration method. Previous studies were believed have limited value to evaluate impact of dosimetric effects of translational and rotational set-up errors because most of them were neither lack of proper method to reestablish rotational set-up errors nor described the effect of rotational set-up errors based on simulation method but few of them using the clinical-obtained set-up errors. Analysis based on simulation data was not realistic and may deduct to an unreasonable conclusion. In this study, a total number of 105 set-up errors were acquired for 10 patients derived before CBCT image registration and correction. A method to correct the 6D set-up changes by adjusting the gantry, collimator, and couch angles of treatment beams (Yue et al. 2006) was used to accumulate dose by fraction in one CT image and evaluate the dosimetric outcome retrospectively. To our knowledge, this is the first time to evaluate target coverage influenced by combined translational and rotational set-up errors as well as target distance away from the treatment center, for IMRT of cervical cancer using online CBCT images with clinical obtained results.

In this study, significant decreace of PTV dose coverage was observed in TplusP plan compared to original plan. The real volume that received prescription dose of PTV is less than 95 % which is the goal suggested by International Commission on Radiation Units and Measurements. ICRU report 62(Prescribing, Recording, and Reporting Photon-Beam Intensity-Modulated Radiation Therapy (IMRT): Contents 2010). Further, we investigated whether target distance away from the treatment center affected dose distribution. The result showed that PTV coverage was significantly correlated with distance away from treatment center, which was in accordance with the report (Laursen et al. 2012). But neither combined translational and rotational set-up errors nor target distance away from the treatment center significantly affected the coverage of CTV. Then, a dramatical phenomenon, coverage of PTV did not achieve the goal of 95 % but coverage of all CTVs maintained a level as high as 98 % which surpassed the assumpted goal that miminun dose to CTV was 95 for 90 % of patients when CTV to PTV margin recipes was defined by van Herk, was observed. We speculated reasons for this dramatical phenomenon were: first, a 6 mm CTV to PTV margin was redundant to account for the positioning errors; second, the CTV to PTV margin recipes (2.5 SD of systematic translational set-up errors plus 0.7 SD of random translational set-up errors) would not be appropriate for IMRT of cervical cancer, especially with the scope of 6D. Due to the function of PTV that ensure dose coverage of CTV, a smaller margin maybe achieve the goal that miminun dose to CTV was 95 for 90 % of patients based on tumor control probability (TCP) model, which should be further studied.

Cervical cancer target always has large cranio-caudal extension. Therefore, rotational uncertainties may potentially cause target displace in particular in the regions furthest away from the treatment center. Published study suggested that margin should increase with distance from the isocenter in order to take rotational errors into account (Laursen et al. 2012). In this study, CTV and PTV were divided into several sections to evaluate the necessity of variant margin recipe. The result showed that PTV coverage was significantly correlated with distance away from treatment center, but it does not affect the coverage of CTV. However, it should be awarded that this result only gave advice for cervical cancer patients without para-aortic irradiation with decent errors distribution. For patients with para-aortic irradiation, target volume was longer in superior-inferior direction, which may potentially be more susceptible due to rotational set-up errors (Zhang and Yu 2009).

To further evaluate the impact of rotational set-up errors alone in dosimetry, dose coverage differences obtained with TplusR plans were compared with Tonly plans. By eliminating rotational set-up errors in dose accumulated plan, dose coverage of target increased as expected. However, result showed that the benefit of rotation corrections was insignificant on CTV and modestly on PTV with high patient variation. This result implied that rotation corrections seem to improve target coverage very little, and a PTV margin of 6 mm was sufficient to take into account residual rotational set-up errors for the majority of patients.

It needs to be mentioned that this study did not consider the influece of organ motion, which is a challenge of IMRT for cervical cancer. Therefore, how to intergrate set-up errors (both translational and rotational) and organ motion to determine PTV margin should be further investigated.

## Conclusions

For cervical cancer IMRT, the dosimetric influence for CTV of the distance away from treatment center and the translational and rotational set-up errors, not corrected after CBCT scanning and image registration was small. A PTV margin of 6 mm was sufficient to take into account geometry uncertainties for most patients. A smaller margin should be further studied with the scope of 6D.
